# Resolution of Electronic States in Heisenberg Cluster Models within the Unitary Group Approach

**DOI:** 10.1021/acs.jctc.2c01132

**Published:** 2023-02-03

**Authors:** Giovanni Li Manni, Daniel Kats, Niklas Liebermann

**Affiliations:** Max Planck Institute for Solid State Research, Heisenbergstr. 1, 70569 Stuttgart, Germany

## Abstract

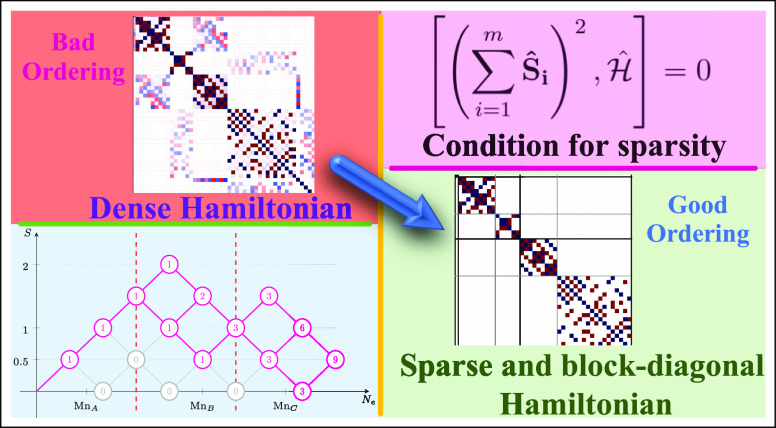

In this work ground and excited electronic states of Heisenberg cluster models, in the form of configuration interaction many-body wave functions, are characterized within the spin-adapted Graphical Unitary Group Approach framework, and relying on a novel combined unitary and symmetric group approach. Finite-size cluster models of well-defined point-group symmetry and of general local-spin  are presented, including *J*_1_–*J*_2_ triangular and tetrahedral clusters, which are often used to describe magnetic interactions in biological and biomimetic polynuclear transition metal clusters with unique catalytic activity, such as nitrogen fixation and photosynthesis. We show that a unique block-diagonal structure of the underlying Hamiltonian matrix in the spin-adapted basis emerges when an optimal lattice site ordering is chosen that reflects the internal symmetries of the model investigated. The block-diagonal structure is bound to the commutation relations between cumulative spin operators and the Hamiltonian operator, that in turn depend on the geometry of the cluster investigated. The many-body basis transformation, in the form of the orbital/site reordering, exposes such commutation relations. These commutation relations represent a rigorous and formal demonstration of the block-diagonal structure in Hamiltonian matrices and the compression of the corresponding spin-adapted many-body wave functions. As a direct consequence of the block-diagonal structure of the Hamiltonian matrix, it is possible to selectively optimize electronic excited states without the overhead of calculating the lower-energy states by simply relying on the initial *ansatz* for the targeted wave function. Additionally, more compact many-body wave functions are obtained. In extreme cases, electronic states are precisely described by a single configuration state function, despite the curse of dimensionality of the corresponding Hilbert space. These findings are crucial in the electronic structure theory framework, for they offer a conceptual route toward wave functions of reduced multireference character, that can be optimized more easily by approximated eigensolvers and are of more facile physical interpretation. They open the way to study larger *ab initio* and *model* Hamiltonians of increasingly larger number of correlated electrons, while keeping the computational costs at their lowest. In particular, these elements will expand the potential of electronic structure methods in understanding magnetic interactions in exchange-coupled polynuclear transition metal clusters.

## 1 Introduction

Symmetry represents a core concept in physics and chemistry, as it helps to dramatically reduce interpretational and computational costs. Translational symmetry in lattices defines its long-range periodic order. Point-group symmetry in crystals and molecules defines the local (point) symmetry, which includes reflections, rotations, and the inversion. The Pauli exclusion principle for Fermionic systems offers another crucial example of the importance of symmetry in electronic structure theory (exchange symmetry). It requires any many-body wave function of a Fermionic system to be antisymmetric with respect to exchange of two particles. This feature has prompted the electronic structure theory community to adopt the Slater determinants as basis to describe the many-body wave functions of multifermionic systems. Resolving the antisymmetry during the optimization of the many-body wave function on an unsymmetrized basis, such as the Hartree products, would represent a major challenge for approximated eigensolvers, both for the much larger optimization space and for the optimization coefficients must perfectly couple across the space to guarantee antisymmetry. Generally, it is not possible to rigorously meet the latter condition via approximated eigensolvers.

Turning our attention toward spin symmetries, it is worth mentioning the spin-projection  preserving symmetry, and the total spin  preserving symmetry, embedded in Slater determinants and configuration state functions (CSFs) bases, respectively. In analogy to Slater determinant bases, which enforce antisymmetry and the spin-projection quantum number by construction, spin-adapted bases enforce total spin symmetry, while reducing the size of the corresponding Hilbert space, limited to the components of the desired total spin. There are multiple ways to create a basis of spin-adapted CSFs.^1−3^ In this work, we use the unitary group approach to spin adaptation in its graphical form (GUGA), pioneered by Paldus^4−7,10,12^ and Shavitt,^8,9,11^ which relies on the generators  and  of the special unitary group of order 2, SU(2), and on the spin-free formulation of the Hamiltonian operators. For example, the spin-free *ab initio* nonrelativistic molecular electronic Hamiltonian within the Born–Oppenheimer approximation reads as
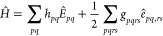
1where  and  are the spin-free excitation operators, and *h*_*pq*_ and *g*_*pqrs*_ the molecular one- and two-body electron integrals.^13^ The GUGA approach is widely utilized within the chemistry community, and it is at the core of many electronic structure methodologies,^14^ including complete/restricted/generalized active space self-consistent field (CASSCF,^15−18^ RASSCF,^19^ GASSCF),^20^ and more recently, GUGA full configuration-interaction quantum Monte Carlo, (GUGA-FCIQMC)^21,22^ and Stochastic-CASSCF.^23^

Discrete symmetry transformations, given by an operator  that commutes with a given Hamiltonian operator, , are pivotal in electronic structure calculations. Commuting operators admit common eigensolutions. Thus, the operator  can be used to filter eigenstates of  with specific eigenvalues of . For example, we may take advantage of the fact that  and  commute, construct the modified  Hamiltonian, and, by tuning the parameter α, control the spectral ordering of the  eigenstates. This strategy has been recently used to gain control over the spin in the Slater-determinant (SD) based full configuration-interaction quantum Monte Carlo (SD-FCIQMC) method to approximate eigenstates of full configuration-interaction (full-CI) quality in large active space calculations, while being able to selectively target states of specific spin.^24^ In virtue of the vanishing commutator the modified Hamiltonian, , shifts the eigenvalues proportionally to *αS*(*S* + 1) while keeping the eigenstates unchanged compared to those of the original Hamiltonian operator, .

Other transformations exist that lead to a modified Hamiltonian  with a unique block-diagonal structure in the transformed basis, and to more compact eigenstates, while keeping the electronic spectrum unchanged compared to the original  operator. These transformations can be described as a *similarity transformation* of the original Hamiltonian,
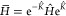
2where  is for example an orthogonal orbital permutation matrix.

We have recently studied the effect of exchanging *orbitals* or *lattice sites* on spin-free *ab initio* and model Hamiltonian matrices and on their eigenstates expressed in a spin-adapted basis.^23,25−28^ Orbital permutations can be described as a 90° rotation between pairs, from which the  matrix is promptly recognized. We found that specific chemically and physically motivated site permutations bring the Hamiltonian matrices into a unique (*quasi*-)block-diagonal structure, and many-body wave functions into embarrassingly compact forms, indicated by larger leading CI coefficients, small *L*_1_-norm and large *L*_4_-norms of *L*_2_-normalized eigenvectors.^25,28^ As a direct consequence of the block-diagonal structure of the Hamiltonian matrices, it is possible to selectively optimize electronic excited states without the overhead of calculating the lower-energy states, by simply relying on the initial *ansatz* for the targeted wave function. This strategy has been numerically shown for the singlet low-energy excited states of two Fe_4_S_4_ cubane clusters.^26^ The block-diagonal structure of the Hamiltonian matrix, the compression of its eigenstates, that emerge in the GUGA Hamiltonian matrix, and the possibility to selectively target specific excited electronic states, represent three additional theoretical advantages (to the best of our knowledge reported by us for the very first time) in employing spin-adapted bases in electronic structure calculations, in addition to the already known advantages of preserving spin symmetry.

Why are such block-diagonal structure and the corresponding wave function compression desirable in quantum chemical simulations of ground and excited electronic states of strongly correlated systems? In quantum chemistry, multiconfigurational approaches are used to generate qualitatively correct wave functions for electronic states of molecules that are not adequately described by single-reference approaches, exemplified by Hartree–Fock, single-reference coupled-cluster and density functional theory methodologies. Exchange-coupled polynuclear transition metal (PNTM) clusters represent a broad class of chemical compounds that are far from being well characterized by single-reference techniques. In multiconfigurational methods, electronic state wave functions are described as linear combinations of electronic configurations, in the form of Slater determinants or spin-adapted functions. Typically multiconfigurational wave functions of PNTM clusters feature multiple dominant coefficients. We refer to those as *multireference* wave functions. The number of electronic configurations defines the configuration interaction (CI) space. The CI space that includes all symmetry allowed configurations within the chosen one-electron basis is referred to as the *full-CI* space. If a subset of the one-electron basis is chosen (the active space) the many-body expansion is referred to as the complete active space (CAS) wave function. The size of full-CI (or CAS) expansion grows exponentially with the number of correlated electrons and one-electron basis functions.^13,14,29^ For Hamiltonian matrices of small dimensions (up to a few thousand configurations), the optimization of the CI expansion coefficients is generally done by exact diagonalization procedures, such as the Jacobi eigensolver for real symmetric matrices. For larger CI problems (approaching a billion many-body functions), Davidson or Lanczos techniques are utilized to compute *few* smallest (or largest) eigenvalues (and eigenvectors).^13,14,30^ For even larger problems, approximate techniques are employed. Density-matrix renormalization group, DMRG,^31−33^ and FCIQMC^21,34,35^ are two examples. While DMRG requires a low entanglement entropy of the ground state to yield accurate results, FCIQMC benefits from sparsity of the ground-state vector. In the latter case, transformations that lead to Hamiltonian matrices with a block-diagonal structure have the obvious advantage of reducing the optimization space to the block of interest.

This paper focuses on how to identify such transformations in Heisenberg Hamiltonian matrices for multisite clusters within the GUGA spin-adapted framework. *Ab initio* Hamiltonian matrices of exchange-coupled PNTM systems, which can be mapped to equivalent Heisenberg models, behave similarly up to the leading terms (quasi-*block*-diagonal structure), as we have numerically shown in earlier works.^26^

The unprecedented wave function compression and block-diagonal structure of the nonrelativistic *ab initio* Hamiltonian matrix in the similarity-transformed spin adapted basis has been discussed in great detail in a number of earlier works of ours, via numerical examples offered by exchange-coupled PNTM clusters, exemplified by iron–sulfur clusters (dimers and cubanes),^23,25,26^ and manganese–oxygen trinuclear molecular systems.^27^ We have also applied this strategy to the one-dimensional s- isotropic Heisenberg model with nearest-neighbor (NN) interactions (single *J* magnetic coupling constant), and to *ab initio* Hamiltonians in the form of chains of equally spaced hydrogen atoms.^28^ For all the cases above we discussed the rationale behind the spin-adapted ground state wave function compression as a function of the permutational symmetry.

While the one-dimensional Heisenberg model with NN interactions is exactly solvable via the Bethe *ansatz*,^37−39^ Heisenberg Hamiltonians with higher dimensions and/or with long-range interactions (already from second NN interactions) and more complex Hamiltonians, such as the *ab initio* nonrelativistic Hamiltonian, remain elusive.^40^ Methods based on the matrix product state paradigm,^41−44^ such as DMRG,^31−33^ are very successful for 1D systems, even with periodic boundary conditions^45,46^ and long-range interactions. For lattice models of higher dimensions, tensor network state approaches have been applied with some success.^47−51^ Also, quantum Monte Carlo procedures have been able to provide accurate numerical solutions as the Heisenberg model can be solved without a sign problem on a bipartite lattice.^52−76^

In the present work, we expand our understanding of the block-diagonal structure of the spin-free many-body Hamiltonian matrices and the related compression of spin-adapted eigensolutions as a function of site permutations. We generalize the compression to ground- and excited-states wave functions of finite-size Heisenberg Hamiltonians, for sites with , and consider more than one magnetic coupling constant. Such models are often used to describe magnetic interactions in biological and biomimetic PNTM clusters with unique catalytic activity, such as the nitrogen fixation and the photosynthesis. The Heisenberg models discussed here exhibit a more complex electronic spectrum as compared to the single-*J* one-dimensional Heisenberg model (chain).

In our previous works a more *phenomenological* approach has been undertaken to explore the compression of the electronic wave functions as a function of the orbital/site reordering. For molecular *ab initio* Hamiltonians of PNTM clusters, including high-valent  trinuclear clusters and iron–sulfur dimers  and cubanes , chemically motivated reorderings were suggested.^23,26,27^ Similarly, for the one-dimensional Heisenberg chain conclusions were obtained following a thorough exploration of the permutational space, also adopting a simulated annealing strategy.^28^ In the present work we undertake a more rigorous and fundamental strategy to the block-diagonal structure of the electronic Hamiltonian within a spin-adapted formulation, based on commutation relations between partial cumulative spin operators and the Hamiltonian operator. These commutation relations represent a new tool to predict orbital/site permutations that lead to wave function compression without necessarily explore numerically the permutational space. The strength of this strategy is its generality and transferability to other model systems and Hamiltonians. The proposed approach greatly enlarges the applicability of wave function-based strategies, allowing for computationally inexpensive and reliable characterizations (and predictions) of the electronic structures and magnetic interactions in the ground and/or excited states of exchange-coupled PNTM clusters.

In Sec. 2 we define the Heisenberg model both in terms of the usual spin operators and in terms of the  spin-free operators. In Sec. 3 we show the effect of the site/orbital reordering on the spin-adapted Hamiltonian matrices and their eigenstates for a few selected systems, including the 2- and 3-sites s- clusters. In Sec. 4 a connection between the block-diagonal structure of the Hamiltonian matrix and commutation relations between partial cumulative spin operators and the Hamiltonian operator is made, that allows us to estimate whether block-diagonal structure is possible and what site ordering is to be chosen in order to reveal this feature. We also derive a generalization of the model Hamiltonians and the corresponding optimal site reordering, that induce the block-diagonal structure of the Hamiltonian matrix for clusters with multiple magnetic centers. Our conclusions are offered in Sec. 5.

## 2 The Heisenberg Model

The quantum Heisenberg model^77−81^ is a long-studied model Hamiltonian, widely used to describe magnetism in solids^82−93^ and molecules.^94,95^ In its general form it reads as
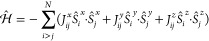
3where the indices *i* and *j* run over all *N* lattice sites,  (with *k* = *x*, *y*, *z*) are the *anisotropic* magnetic coupling constants and  are the components of the local (per site) spin operators. In the NN Heisenberg model, the sum is only performed over neighboring sites ⟨*ij*⟩. The main focus of this work is the isotropic Heisenberg model, for which the Hamiltonian reads as
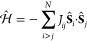
4where the  are the local (per site) spin operators corresponding to the local quantum number . In the previous work,^28^ we focused on the single-J s- Heisenberg model with *isotropic* antiferromagnetic NN interactions, *J*_*ij*_ = *J* < 0
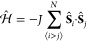
5

For spin-1/2 particles the second-quantized spin-free representation of the scalar product  reads
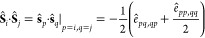
6For the more general case of *S*_loc_ > 1/2, eq 6 becomes
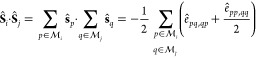
7where *S*_loc_ is the coupled spin at each site, obtained as sum of spin-1/2 vectors  located at lattice site *i* and  at site *j*, respectively.  is the set of all electron indices at site *i*. The condition that  for *i* ≠ *j* is implied. Inserting eq 7 (or similarly eq 6) into eq 4 allows us to express the Heisenberg Hamiltonian in terms of the spin-free excitation operators:^13,96−98^
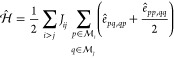
8

Notably, the operator  in eq 8 provides nonvanishing contributions only to the diagonal elements of the Heisenberg Hamiltonian, for (1) δ_*pq*_ = 0 (because ), and (2) considering that the Heisenberg model consists of singly occupied sites, only one term in  can contribute for each function the operator acts on. Consequently,  equals 1 for each *interacting* (*p*, *q*) pair, and it represents a constant shift in the spin-free formulation of the Heisenberg Hamiltonian, which consequently only couples CSFs by pure exchange interactions via the  operator. Equation 7 represents the link between the Heisenberg Hamiltonians and the ab initio molecular Hamiltonian used in quantum chemistry. For systems with more than one electron per site, states with variable *S*_loc_ populate the Hamiltonian matrix. However, for chemical complexes featuring weak ligand-field effects, non-Hund states characterize the higher portion of the electronic spectrum, while for the low-energy electronic states electrons at each site couple to maximize the local spin (Hund states). In model Heisenberg Hamiltonians, non-Hund states can be pushed at the higher end of the energy spectrum by adding an effective ferromagnetic interaction *J*_Hund_ > 0 between electrons residing on the same site. This term is to be added to eq 8. It is relevant to stress that within the Heisenberg model only singly occupied orbitals have been considered (unpaired electrons); thus, there are no configurations coupled via  excitations (a geminal excitation).

In the following, we will investigate in greater detail the block-diagonal structure of Heisenberg Hamiltonian matrices and the compactness of its eigenstates for triangular clusters of isosceles (*C*_2*v*_ point group symmetry), equilateral (*D*_3*h*_) and scalene (*C*_*s*_ point group) symmetry, and 4-site clusters of various point group symmetries. The isotropic Heisenberg Hamiltonian for the isosceles triangle is written as
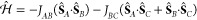
9where the *J*_*BC*_ = *J*_*AC*_ equality applies. This model Hamiltonian has been used to describe magnetic interactions in Mn_3_O_4_ clusters.^27^ For a scalene triangle *J*_*AB*_ ≠ *J*_*BC*_ ≠ *J*_*AC*_ and the Hamiltonian reads as
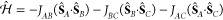
10while for an equilateral triangle *J*_*AB*_ = *J*_*BC*_ = *J*_*AC*_. The one-dimensional 3-site chain with periodic boundaries is identical to the equilateral triangle, and the one with open boundaries is topologically identical to the isosceles triangle Heisenberg Hamiltonian with *J*_12_ = *J*_23_ and *J*_13_ = 0. For a square lattice (*D*_4*h*_ point group symmetry) two nonequivalent magnetic coupling constants exist: a 4-fold *J*_s_ (short) corresponding to the edges of the square (AB, BC, CD, and DA) and a 2-fold *J*_l_ (long) corresponding to the diagonal interactions (AC and BD). In this case the Heisenberg Hamiltonian is given by the following expression
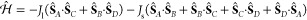
11This Hamiltonian has been used to describe magnetic interactions in Fe_4_S_4_ cubane clusters.^26^

## 3 Permutation Effects on Spin Adapted Basis

In earlier works we have shown the compression effects on ground state wave functions for *ab initio* Hamiltonians^25^ and for the s- one-dimensional Heisenberg model.^28^ In the case of the Fe_4_S_4_ cubane model we have also numerically shown the unique block-diagonal structure of the *ab initio* Hamiltonian matrix that emerges from specific sites/orbitals reordering, and the possibility to selectively target excited states within the same total spin sector and differing in the intermediate spin coupling.^26^ In this section, the effect of the orbital/site reordering and the subsequent wave function compression of ground and excited eigenstates is explored for two Heisenberg cluster models, namely, (a) a two-site cluster with local s- spins, and (b) a 3-site s- cluster, in isosceles triangle geometry. Generalizations to different sizes (multisite clusters) and different topologies (isosceles, equilateral, scalene triangles) are discussed in the next section.

The full Hilbert space size for an electronic system in a spin adapted basis is provided by the Weyl-Paldus dimension formula^4^
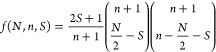
12where *N*, *n*, and *S* refer to the number of correlated electrons, orbitals, and the targeted total spin quantum number (*S* = 0 for singlet, *S* = 1 for triplet, and so on), respectively. However, in the Heisenberg model electrons are not permitted to pair in the same orbital, and charge-transfer states obtained via hopping are not considered. Thus, the configurational space consists solely of configurations commonly known as *spin flips*. The size of the Heisenberg configurational space is provided by the van Vleck–Sherman formula^99^
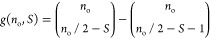
13where *n*_0_ refer to the number of singly occupied (open) sites.

**Figure 1:**
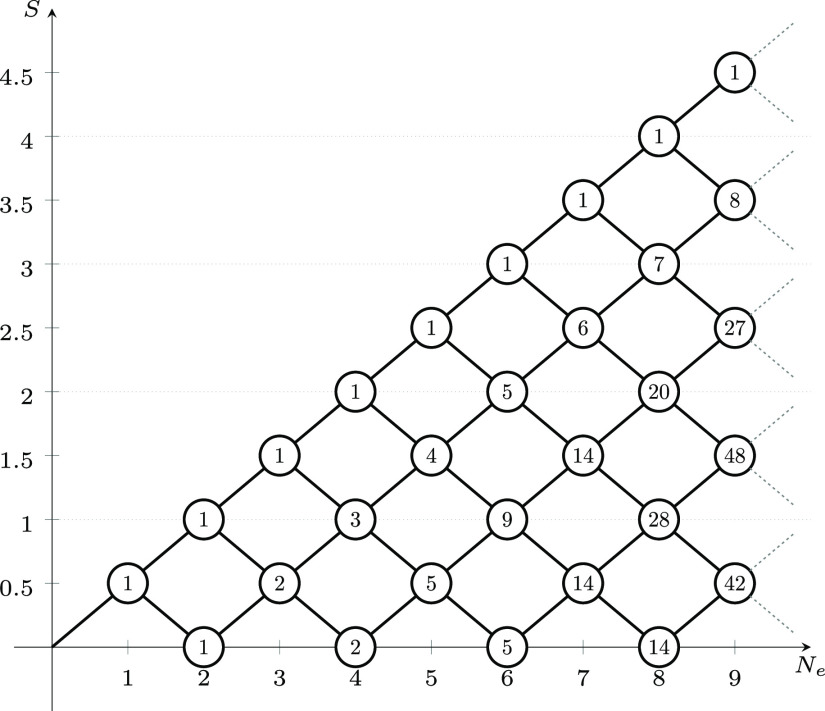
Generic genealogical branching diagram for up to 9 electrons (*N*_e_). The node weights represent the number of paths starting from the *root node*, (*N*_e_, *S*_tot_) = (0, 0) to reach the targeted node. This number is given by the van Vleck–Sherman formula, eq 13.

The spin-adapted basis for any Heisenberg systems can be graphically represented as paths branching through the *genealogical branching diagrams* (see Figure 1).^1,13,14,100^

In these diagrams starting from the *root node* (origin of the graph), electrons are *cumulatively* spin-coupled, contributing positively (up-spin, **u**, ) or negatively (down-spin, **d**, ) to the spin. Thus, for a six-electron system a possible spin-adapted electronic configuration is written as |*uududd*⟩, where the first 2 electrons are positively spin-coupled contributing to the partial cumulative spin *s* = 1; the next electron lowers the partial cumulative spin to ; the next three electrons further spin couple leading to the final *S* = 0. In the hypothetical two-site system with local s- spins, considering that the first 3 and last 3 singly occupied orbitals reside on site *A* and *B*, respectively, the |*uud*, *udd*⟩ CSF is interpreted as follows: the first three unpaired electrons on the magnetic center *A* are coupled to a doublet (violating Hund’s rule), and the other 3 electrons on site *B* are antiferromagnetically aligned to the spin on *A*, thus leading to the total spin *S* = 0. The CSF strings are not to be confused with *m*_*s*_ conserving basis, such as the SDs; in fact, each CSF can be expanded into a linear combination of SDs spanning the same orbital/site space, for example as discussed by Grabenstetter.^101^

### Two-Site s- Model

3.1

**Figure 2:**
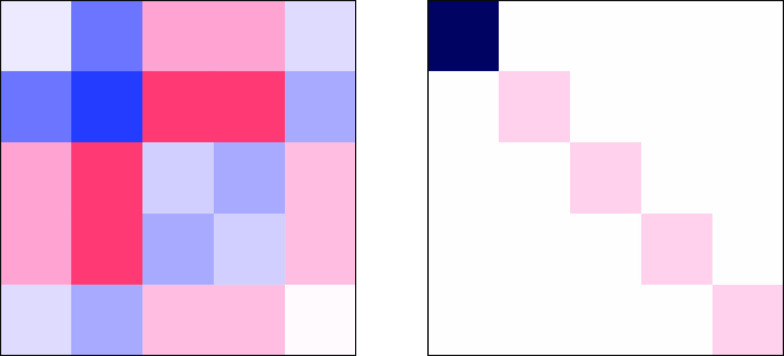
Heisenberg Hamiltonian matrices (*S* = 0) for a 2-site s- system in the GUGA spin-adapted basis and using an arbitrary ordering (1_*A*_ – 2_*B*_ – 3_*B*_ – 2_*A*_ – 3_*A*_ – 1_*B*_) (left) and the *site-separated* ordering (1_*A*_ – 2_*A*_ – 3_*A*_ – 1_*B*_ – 2_*B*_ – 3_*B*_) (right). Red and blue colors refer to elements of opposite sign.

We first consider the ground and excited states of singlet spin symmetry, for a two-site s- Heisenberg model. The system consists of six electrons. On each site parallel spin alignment is favored (large on-site *J*_Hund_ > 0), while keeping an antiferromagnetic interaction *J*_AB_ < 0 across the sites. For the singlet spin symmetry sector the basis of spin-adapted functions consists of 5 CSFs, namely, |*uuuddd*⟩, |*ududud*⟩, |*uuddud*⟩, |*uduudd*⟩, and |*uududd*⟩. These CSFs can be identified as branches in Figure 1. Two orbital orderings are considered, one, where electrons from the two sites are *nonsite-separated* (1_*A*_ – 2_*B*_ – 3_*B*_ – 2_*A*_ – 3_*A*_ – 1_*B*_), and the *site-separated* ordering (1_*A*_ – 2_*A*_ – 3_*A*_ – 1_*B*_ – 2_*B*_ – 3_*B*_). The Hamiltonian matrices in the two orderings are represented in Figure 2.

Strikingly, in the *site-separated* ordering the Hamiltonian matrix is already in diagonal form. A similar feature was already observed for the N_2_ and the Cr_2_ molecules at stretched geometry and using a nonrelativistic *ab initio* Hamiltonian.^25^ The ground state as well as all excited states are inherently single-reference, with only one CSF completely characterizing the many-body wave function. In particular the ground state is fully characterized by the |*uuuddd*⟩ CSF, that is promptly interpreted as two s- local spins coupled antiferromagnetically. In the nonsite-separated ordering the matrix is dense indicating the multireference character of the eigenstates when this particular ordering is chosen. Thus, through a simple process of site/orbital reordering, a diagonal Hamiltonian is obtained, graphically shown in Figure 2, which corresponds to highly compressed eigenvector, to the limit of single-reference wave functions.

### 3-Site s- Model

3.2

In this section we consider the s- Heisenberg model of a 3-site cluster in the isosceles triangle topology, with *J*_*BC*_ = *J*_*AC*_ ≠ *J*_*AB*_.

Combining two spin angular momenta with local spin *S*_local_ = 3/2 results in four intermediate spin states, *S*_interm_ = Γ^(3/2)^ ⊗ Γ^(3/2)^ = Γ^(3)^ ⊕ Γ^(2)^ ⊕ Γ^(1)^ ⊕ Γ^(0)^. The resulting intermediate spins, *S*_interm_, further couple to the third local spin, *S*_local_ = 3/2, leading to 12 spin states (see eq 14).
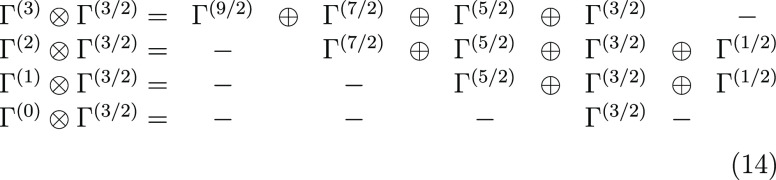


These states range from *S*_total_ = 9/2 to *S*_total_ = 1/2. For *S*_interm_ = 3, the spins on the first two centers are *collinear* parallel and the third center can further couple in a collinear manner, leading to *S*_total_ = 9/2, with all spins parallel aligned, and *S*_total_ = 3/2, with the spin on the third center antiparallel with respect to *AB*. Similarly, for *S*_interm_ = 0 the first two centers show collinear antiparallel spins, while the third center is left uncoupled. The remaining 9 spin states are characterized by noncolinear spin couplings.

**Figure 3:**
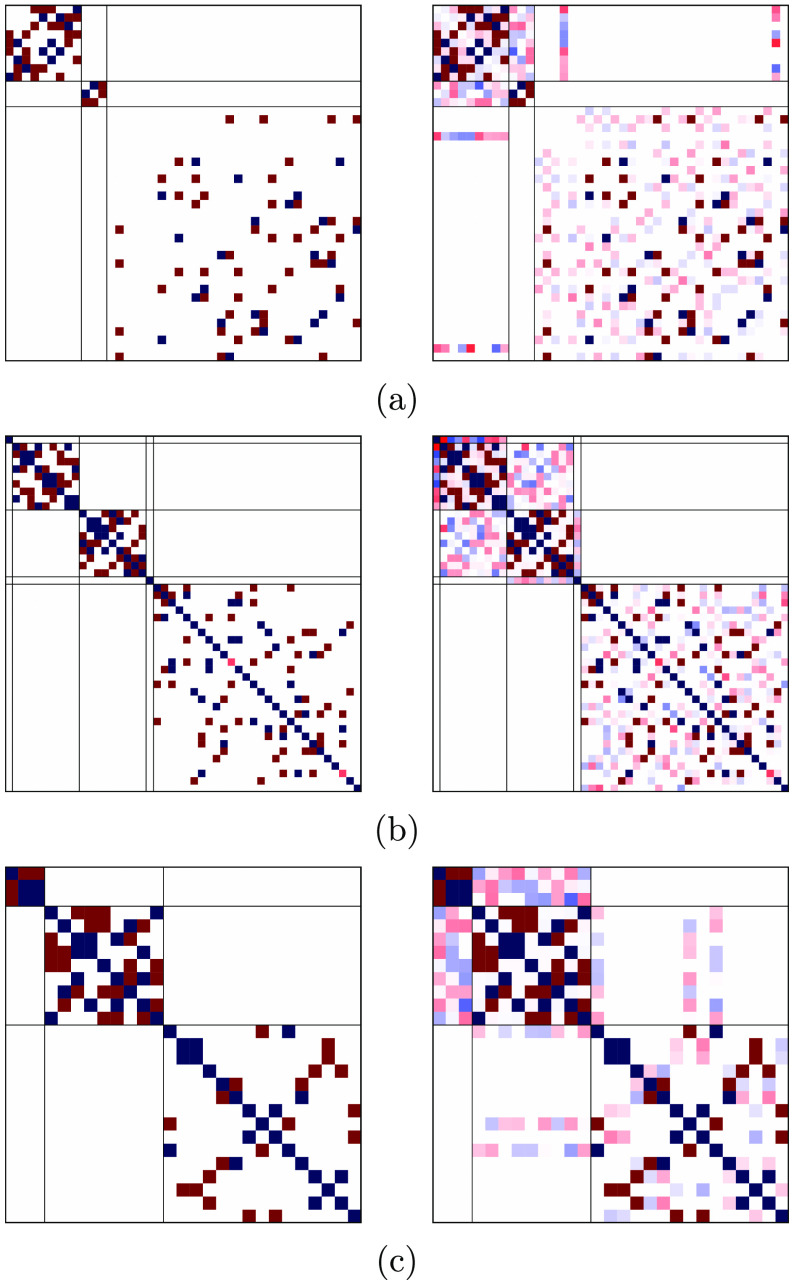
Heisenberg Hamiltonian matrices for a 3-site s- system in the GUGA spin-adapted basis for (a) *S*_total_ = 1/2, (b) *S*_total_ = 3/2, and (c) *S*_total_ = 5/2 total spin symmetries. The *ABC* (*left*) and *ACB* (*right*) orderings have been considered. Red and blue colors refer to elements of opposite sign.

The size of the Heisenberg spin-adapted basis for each of the possible total spin states is given by eq 13 and graphically reported in Figure 1. For a total of 9 unpaired electrons 42, 48, 27, 8, and 1 CSFs form the bases for *S*_total_ = 1/2, 3/2, 5/2, 7/2, and 9/2, respectively. In Figure 3 the Hamiltonian matrices in the GUGA spin-adapted basis are reported for the three largest spin symmetries, namely, *S*_total_ = 1/2, 3/2, and 5/2.

In Sec. 3.1 we have shown that *site-separated* orbital orderings lead to maximal compression of the GUGA wave function. For the 3-site case we adopt the same strategy. However, for the 3-site problem the site-permutation degree of freedom are also to be addressed. Of the 3! = 6 possible site permutations (*ABC*, *ACB*, *BAC*, *BCA*, *CAB*, *CBA*) only permutations that are nonequivalent by symmetry are retained, namely, *ABC* and *ACB*. Notably, *ACB* and *CAB* orderings are equivalent because the interactions between the first two sites, *AC* or *CA*, and the last site, *B*, are identical by symmetry.

In Figure 3 we see an important difference between the *ABC* (left) and the *ACB* (right) orderings. A clear block-diagonal structure emerges for the *ABC* site ordering that is largely lost for the *ACB* ordering. In the following we will analyze in greater details the Hamiltonian matrices of the quartet spin state, Figure 3b. Moving from the upper-left part of the Hamiltonian matrix in *ABC* ordering 4 blocks can clearly be distinct from the rest of the matrix. All four blocks share a common feature, that is the local spin expectation value . Next, CSFs with common well-defined cumulative  value characterize the 4 distinct blocks. The first block only contains the |*uuu*, *ddd*, *uuu*⟩ CSF with . The second block contains CSFs with , such as |*uuu*, *ddu*, *duu*⟩ and |*uuu*, *dud*, *duu*⟩. The third block contains CSFs with , such as |*uuu*, *duu*, *udd*⟩, and the fourth block contains solely the |*uuu*, *uuu*, *ddd*⟩ CSF with intermediate . The remaining CSFs that populate the fifth block are CSFs that violate the local Hund’s rule already on the first site, Thus, , which is obtained for all those CSFs starting with |*uud*...⟩ or |*udu*...⟩. Sparsity is also observed in the non-Hund block.

On the right-hand side of Figure 3, for which the *ACB* ordering is utilized, nonvanishing matrix elements populate the off-diagonal blocks. However, the block-separation between Hund-states and non-Hund states holds even in the less-optimal *ACB* ordering. This feature is a direct consequence of retaining the site-separated orbital list in the *ACB* site ordering.

A similar block-diagonal structure has already been reported for nonrelativistic *ab initio* Hamiltonians applied to trinuclear Mn_3_O_4_ clusters.^27^

The block-diagonal structure in the *ABC* site-ordering leads to an extraordinary compression of the ground- and excited-state many-body wave functions. Additionally, it allows to selectively target excited states, exclusively relying on the initial wave function *ansatz*. In the example above, choosing the |*uuu*, *ddu*, *duu*⟩ CSF as trial wave function unequivocally leads to the lowest electronic state of the second block, a state with . This strategy has already been employed for *ab initio* Hamiltonians of Fe_4_S_4_ cubane systems, featuring local spin .^23,26^ In the case of the less-optimal *ACB* ordering it is not possible to separate states with different intermediate spin coupling , due to the presence of the off-diagonal blocks, thus in general any choice of trial wave function inevitably leads to the ground state wave function, preventing any selective optimization of excited states.

**Figure 4:**
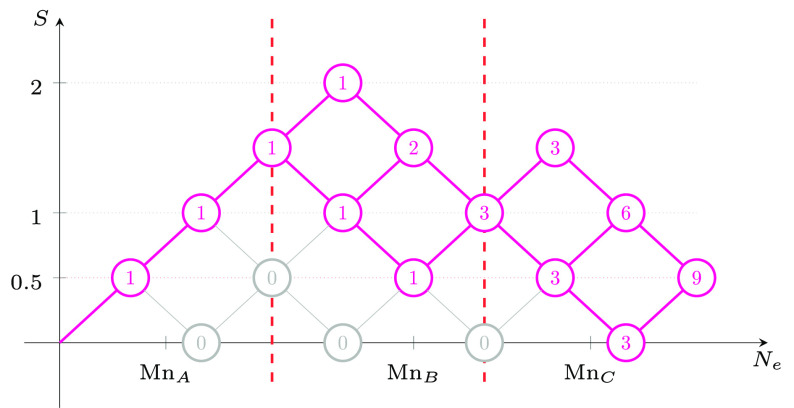
Genealogical branching diagram for 9 electrons distributed over 3 magnetic sites (Mn_*A*_, Mn_*B*_, and Mn_*C*_). The paths compatible with *S*_*A*_ = 3/2 (local Hund state on the Mn_*A*_ site) and  are highlighted in magenta. These paths correspond to the 9 CSFs of the *S*_tot_ = 1/2 state with a  intermediate spin coupling.

Interestingly, the size of the blocks obtained in the *ABC* ordering can be anticipated by means of the genealogical branching diagrams. In Figure 4 the branching diagram of 9 electrons coupled to a doublet spin state is reported, under the constraint . There are precisely three paths that lead to  while preserving the local spin on site A (*S*_*A*_ = 3/2 in this example). Three more paths exist for the coupling with the *C* site. Thus, a total of nine paths and equivalent CSFs are the only possible for this spin-state. Similar arguments can be utilized to identify the basis contributing to the different blocks of the Hamiltonian matrix. Figure 4 represents a concrete measure of the minimal multireference character of electronic states in an optimal site ordering and within the GUGA spin-adapted basis.

## 4 Block-Diagonal Hamiltonians and Commutators

While in Sec. 3 we collected examples of model Hamiltonians exhibiting a block-diagonal structure, and analyzed in detail the structure of those matrices and their eigensolutions, in this section we provide a more rigorous rationale for the emerging of such unique and computationally advantageous matrix structures, which complement the chemically/physically motivated site reorderings and the simulated annealing strategy to identify the optimal site permutations.^28^

For this, we turn our attention to commutator relations between cumulative spins and the Heisenberg Hamiltonians. Commuting operators, , admit common eigensolutions. Thus, in a basis of eigenfunctions of , the matrix  has a block-diagonal structure according to the degenerate eigenvalues of .

For a two-site system, the Heisenberg Hamiltonian is proportional to the  operator. Because of the commutation relation
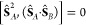
15(see Appendix A.1 for a proof), on the basis of eigenfunctions of ,  and therefore the corresponding Heisenberg Hamiltonian are blocked according to different values *S*_*A*_(*S*_*A*_ + 1). In the *site-separated* orbital ordering, each CSF represents an eigenfunction of the cumulative partial spin operators , , , and so on, and they can certainly be separated according to the  value. In the *non-site-separated* orbital ordering each individual GUGA CSF is not an eigenfunction of . From this, the dense structure of the Hamiltonian matrix and of the resulting eigensolutions follow.

From eq 15 it follows that
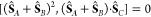
16and
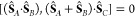
17(see Appendix A.2 for an alternative proof). Therefore, as already shown for the two-site case, any eigensolution of the isosceles triangle Heisenberg Hamiltonian, is also an eigensolution of the partial cumulative spin  (or, in the case of degeneracies, can be cast in this form). In the *ABC* ordering, CSFs with a common  expectation value, will form blocks in the Hamiltonian matrix, orthogonal to the other blocks. When the *ACB* ordering is chosen, CSFs only form an eigenbasis of the cumulative , which does not commute with the Hamiltonian. Thus, operating on an intermediate eigenbasis of  does not bring any advantageous blocking structure, as opposed to the *ABC* case.

The relation between commuting operators and block-diagonal structure for the isosceles triangle can trivially be extended to the 3-site chain with open boundaries (a special case with *J*_*AB*_ = 0) and the equilateral triangle (another edge case with *J*_*AB*_ = *J*_*BC*_ = *J*_*AC*_).

**Figure 5:**
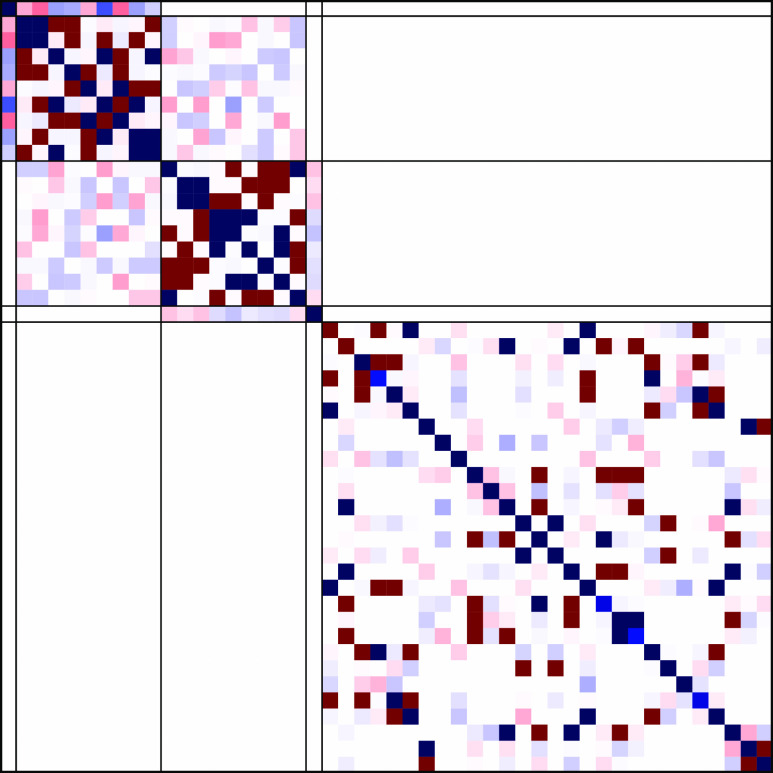
Hamiltonian matrix for a 3-site *s*-3/2 Heisenberg system with three nonequivalent magnetic coupling constants (*J*_*AB*_ ≠ *J*_*BC*_ ≠ *J*_*AC*_). Red and blue colors refer to elements of opposite sign.

**Figure 6:**
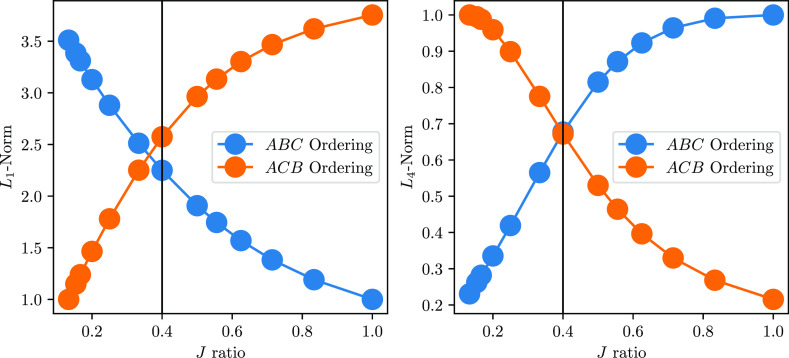
*L*_1_-norm (left) and *L*_4_-norm (right) of the lowest quartet spin state for the 3-site s-3/2 Heisenberg Hamiltonian with fixed *J*_*ab*_ = −150 and *J*_*ac*_ = −20 values and variable *J*_*bc*_, spanning the [−20, −150] range (arbitrary units). The *J*_*ac*_/*J*_*bc*_ ratio is used for the *x*-axis. Lower values of the *L*_1_-norm and higher values of the *L*_4_-norm are associated with a more compressed wave function. The vertical black line at *J*_*ac*_/*J*_*bc*_ = 0.4 (corresponding to *J*_*bc*_ = −50) marks the compression flipping point. For *J*_*ac*_/*J*_*bc*_ < 0.4 the ACB ordering and for *J*_*ac*_/*J*_*bc*_ > 0.4 the *ABC* ordering are to be preferred, respectively.

We stress here that  commutes with the sum of  and  but not with individual terms. Precisely for this reason, no commutation relations exist for a scalene triangle (*J*_*AB*_ ≠ *J*_*BC*_ ≠ *J*_*AC*_), except the commutator of local spin  with the Hamiltonian. Thus, the block-diagonal structure that separates states with different partial cumulative spin  is not present for the scalene triangle, but the block-diagonal structure over  (Hund and non-Hund states) remains. This finding is also shown graphically in Figure 5. This matrix should be compared to the one reported in Figure 3b (left). The value of the off-diagonal matrix elements for the scalene triangle is proportional to the (*J*_*BC*_ – *J*_*AC*_) difference. The closer *J*_*BC*_ and *J*_*AC*_, the more the off-diagonal elements become vanishingly small. In these cases the quasi-*block*-diagonal structure, albeit not exact, allows partial compression (in the numerical sense of increasing the *L*_1_-norm), which is beneficial for methods that approximate the full-CI wave functions, such as FCIQMC,^21,34,35^ as it enhances the numerical stability of the eigensolver. Also, as we have observed for the isotropic one-dimensional Heisenberg model,^28^ the leading CSFs already carry the most important forms of (long-range) electron correlation, even though they are numerically not converged to the exact solution. The dependency of the compression with respect to the deviation from the isosceles triangle topology is illustrated in Figure 6 for two different reorderings.

**Figure 7:**
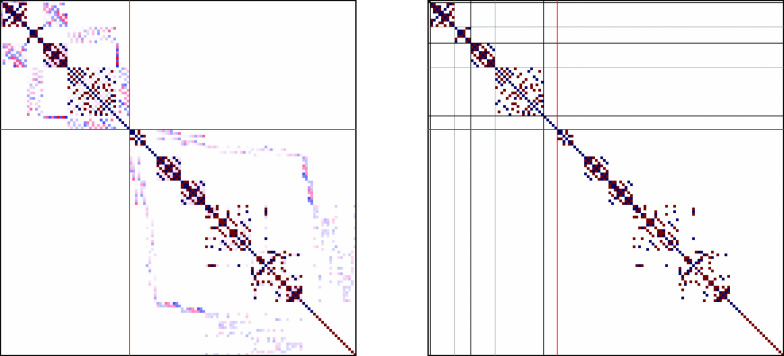
Heisenberg Hamiltonian matrix for a 4-site s- square cluster, with two J magnetic coupling parameters, *J*_*AB*_ = *J*_*BC*_ = *J*_*CD*_ = *J*_*DA*_ = *J*_short_ and *J*_*AC*_ = *J*_*BD*_ = *J*_long_, in the GUGA spin adapted basis, for a singlet spin state (*S*_tot_ = 0). The *ABCD* (left) and *ACBD* (right) site orderings have been considered. The *ACBD* ordering ensures a bock-diagonal structure of the matrix with eigenbasis of the cumulative  with common eigenvalues grouped together. In the *ABCD* ordering such block-diagonal structure is partially lifted. Red and blue colors refer to elements of opposite sign.

With the commutation relations discussed above it is possible to make predictions on more complex model systems containing a larger number of magnetically coupled sites. In the following we consider the Heisenberg Hamiltonian for a  4-site square cluster, eq 11. Using the commutation relation
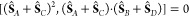
18it is easy to demonstrate that  commutes with the Heisenberg Hamiltonian in eq 11. Equation 18 suggests that in the *ACBD* ordering CSFs with common partial spins  eigenvalues group together forming a block-diagonal structure of the full Hamiltonian matrix. This result is numerically confirmed in Figure 7.

This finding explains on a rigorous ground, what we have numerically shown for the iron–sulfur cubanes in an earlier work.^26^ The same argument justifies the block-diagonal structure and corresponding wave function compression in the 4-site chain with periodic boundaries (extreme case of a 2*J* Heisenberg model with *J*_*l*_ = 0). When *open boundaries* are considered the commutation relation of eq 18 does not hold, and eigensolutions of the Hamiltonian are no longer eigensolutions of partial spin operators, and a denser Hamiltonian is to be expected.

The commutation relations found above can also be proven using the language of second quantization. To that end we write the local spin operator (per site) as
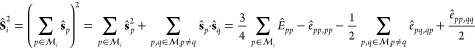
19and utilize eq 7 for the spin–spin correlation operator .

### 4.1 Cumulative-Spin-Blocked Heisenberg Hamiltonian

A general expression of a *n*-site Heisenberg Hamiltonian can be derived, which in the spin-adapted GUGA framework features a block diagonal matrix structure for *all* cumulative spins, , , , and so on. The cumulative spin of the first *m* sites can be expressed as
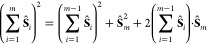
20Using eq 17 it can be easily shown that a product of the cumulative spin with the spin of another site commutes with any other product of any cumulative spin with another site,
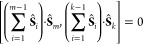
21Finally, we introduce the following *n*-site Heisenberg Hamiltonian,
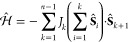
22which commutes with all cumulative spins calculated in the same order, as can be demonstrated by utilizing eqs 20 and 21.

**Figure 8:**
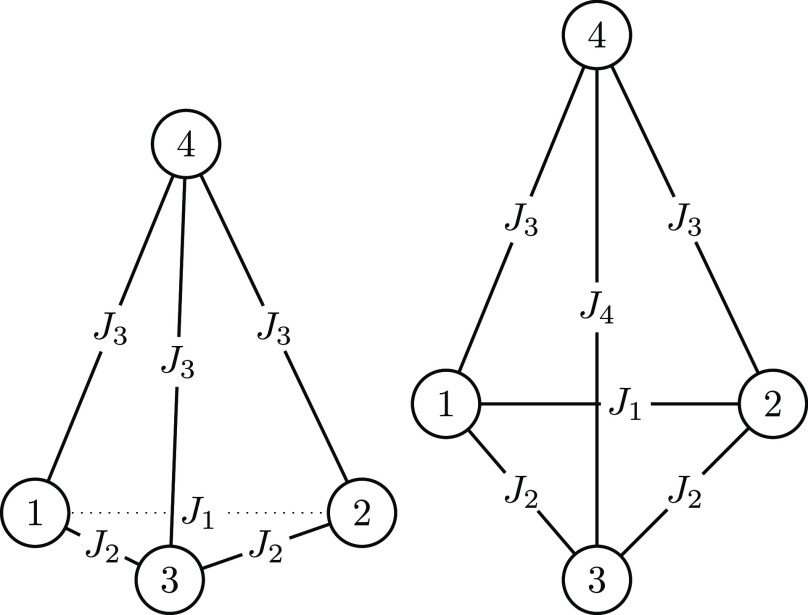
4-site Heisenberg model with the optimal cumulative-spin blocking (left), and with the blocking only according to the cumulative spin of the first two sites (right).

Examples for this type of system are the isosceles triangle, *vide supra*, and a 4-sites structure as depicted in Figure 8 (left)
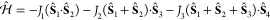
23

Instead of enforcing commutation of the model Hamiltonian with *all* cumulative spins (eq 22), another Hamiltonian can be introduced that commutes only with the first *m* cumulative spins (a less strict requirement),
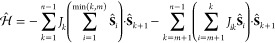
24The 4-site square model Hamiltonian is a special case of eq 24, for which only blocking according to the cumulative spin of the first two sites is assured. A more general example of a 4-site cluster that features blocking only up to a certain level of cumulative spin is depicted in Figure 8 (right).

## 5 Conclusions

In this work we have described in great detail a novel combined symmetric and unitary group approach that yields a unique block-diagonal structure of the many-body Hamiltonian matrix for general Heisenberg cluster models. As a consequence of the block-diagonal structure, more compact ground- and excited-state wave functions are obtained. This compression arises from well-defined *ordering* of the molecular orbitals and sites, combined with the GUGA cumulative spin coupling. We demonstrate that molecular orbital (and site) ordering is bound to specific commutation relations between cumulative spin operators and the Hamiltonian operator.

In the compressed many-body wave functions a greatly reduced number of spin-adapted electronic configurations (CSFs) is necessary to characterize the electronic structure of the targeted state, to the limit of single reference wave functions (one CSF). The wave function compression greatly facilitates the convergence of methods that approximate the full-CI solutions, such as the spin-adapted GUGA-FCIQMC^21,22^ approach, as their accuracy strongly depends on the sparsity of the Hamiltonian and its eigensolutions. Moreover, the block-diagonal structure of the Hamiltonian allows direct *state-specific* wave function optimizations of ground and excited states, while removing the undesired overhead of computing all states energetically more stable than the targeted one. This framework is of general applicability. While in this work we have used Heisenberg cluster models, and explained in greater detail the role of commutation relations, we have observed equivalent compressions in ground- and excited-state wave functions of exchange-coupled PNTM clusters, such as the , , and  clusters.^23,25−27^ The strategy has successfully been applied also to other model Hamiltonians (one-dimensional s-1/2 Heisenberg model) and their *ab initio* equivalent (chain of equally spaced hydrogen atoms).

While the previous works were based on general chemical/physical considerations and partially automated techniques (simulated annealing)^28^ to identify the optimal ordering, in the present work we show that a *sufficient* condition exists to predict at the most fundamental level the optimal site ordering: *Block-diagonal Hamiltonian matrices and highly compressed eigenvectors are obtained for site orderings that make the cumulative spin and the Hamiltonian operators commute.* For example, eq 15 suggests that it is possible to find solutions to the 2-site Heisenberg model that are also solutions of the local  operator. And considering that in site-separated orbital ordering, CSFs are already eigensolutions of the cumulative local spin operators, CSFs with different local spin eigenvalues do not mix, nor they will mix via the Heisenberg Hamiltonian (due to eq 15), from which the block diagonal structure arises. Similar commutation relations have been discussed for tri- and tetra-nuclear cluster models, showing also the differences that emerge from different topologies (isosceles, equilateral, scalene triangles). For each case, the commutation relations between partial cumulative spin operator and Hamiltonian suggest the optimal site-ordering. A generalization of these commutation rules has been derived. PNTM clusters with topologies matching the one suggested by our general commutation rules are to be expected in nature. For those clusters our strategy offers the best possible ordering for the most compact wave function representation for ground and excited states. The commutation relations represent a *sufficient* condition, thus it is possible to observe compressions and block-diagonal structures also in cases where the above commutation relations are not fulfilled. Finally, it is important to realize, as shown in Figure 6, that if deviations from the ideal topology exist for the cluster model investigated, one may still experience wave function compression and a *quasi*-block diagonal structure of the Hamiltonian. This situation in still highly advantageous for methods that approximate the exact full-CI solutions, as the most important correlation effects are already contained in the few leading electronic configurations. The main practical target of our strategy is the computational study and fundamental understanding of magnetic interactions in exchange-coupled PNTM clusters occurring in nature or the corresponding biomimetic counterparts, such as the manganese cluster in photosystem II, the active sites of the nitrogenases, or the synthetic  complex. The extension of this strategy to other classes of chemical systems is currently under investigation and we do not exclude its application to transition metal complexes featuring strong interactions with noninnocent ligands.

## Appendix: Proof of Commutation Relations

### A.1 Two-site case

We want to show that

25

We start by writing the scalar products explicitly as

26

All summations in this section run over the Cartesian coordinates (*i*, *j*, *k* ∈ {*x*, *y*, *z*}). Using the commutation relation

27

we can rewrite this as

28

The other two terms vanish because they only contain commutators of operators that solely act on different sites, which are zero. We then input the usual commutation relation for angular momentum operators

29

where ϵ_*ijk*_ is the Levi-Civita symbol. This leads to

30

In the last three lines the indices in the second term were first renamed and then reordered to restore the ordering *ijk* in the Levi-Civita symbol which leads to sign change.

### A.2 Three-Site Isosceles Triangle

With a similar argument, we can prove that

31

For this, we split up *c*_*AB*,*AC*+*BC*_ into *c*_*AB*,*BC*_ and *c*_*AB*,*AC*_ and again utilize eq 27. This time, three of the four terms vanish for each of the commutators. This leads to

32

Again, by renaming and reordering *i* and *k* in the second commutator, we can write the sum as
